# Preoccupied attachment style and beliefs about medicines in patients with Major Depressive Disorder

**DOI:** 10.1192/j.eurpsy.2022.1425

**Published:** 2022-09-01

**Authors:** G. Santarelli, G. Sanfilippo, F. Benvenuti, L. Santoro, A. Nistri, A. Ballerini, V. Ricca

**Affiliations:** 1 University of Florence, Human Health Sciences, Firenze, Italy; 2 University of Florence, Human Health Sciences, firenze, Italy

**Keywords:** attachment style, beliefs about medicines, major depressive disorder, depressive disorders

## Abstract

**Introduction:**

Attachment style is defined by the American Psychological Association as “the characteristic way people relate to others in the context of intimate relationships”. Four attachment styles have been described: secure, fearful, preoccupied, and dismissing. While the effect of attachment style on psychotherapy was widely investigated, few studies have investigated its role in determining beliefs about medicines in patients with Major Depressive Disorder (MDD).

**Objectives:**

This study aimed to investigate the relationship between preoccupied attachment style and beliefs about medicines in patients with MDD.

**Methods:**

27 patients admitted in the Psychiatric Unit of Careggi with diagnosis of MDD were enrolled. Working Alliance Inventory - patient version (WAI-P), Relationship Style Questionnaire (RSQ) and Beliefs about Medicines Questionnaire (BMQ) were administered. An ANCOVA model having BMQ total score as dependent variable and age, sex, RSQ preoccupied attachment subscale and WAI-P task subscale as predictors was considered. WAI-P task was intended to assess the role of agreement on therapeutic choices.

**Results:**

The overall model was significant (F(4,22)=9,571, P<0.001) and explained 66.8% of BMQ total score variance (R^2^=0.668). Both RSQ preoccupied attachment subscale (B=3.331, t(22)=3.907, p=0.001) and WAI-P task subscale (B=0.238, t(22)=4.565, p<0.001) showed a positive correlation with BMQ total scores. RSQ preoccupied attachment subscale explained 44.6% of variance of BMQ total scores (partial η^2^=0.446), WAI-P task explained 52.3% of variance of BMQ total scores (partial η^2^= 0.523). Age (B=0.059, t(22)=1.588, p=0.129) and sex (F(1,22)=0.035, p=0.854) had no significant effect.

**Figure:**
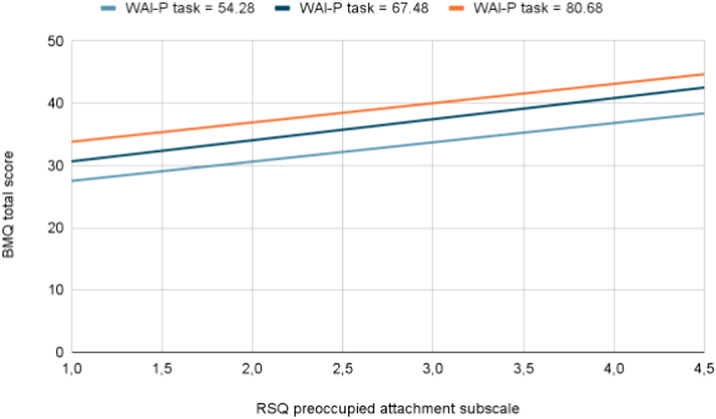

**Conclusions:**

These preliminary data suggest a possible influence of preoccupied attachment style on beliefs about medicines in patients with MDD.

**Disclosure:**

No significant relationships.

